# The effect of managed transition on the proportion of young people transitioning from CAMHS to AMHS: Analysis of the Milestone Cluster Randomised Clinical Trial

**DOI:** 10.1017/S0033291726103766

**Published:** 2026-04-06

**Authors:** Tobias Rowland, Catherine Winsper, Dafne Morroni, Rebecca Appleton, Helena Tuomainen, Gwen C Dieleman, Tomislav Franić, Giovanni de Girolamo, Jason Madan, Athanasios Maras, Fiona McNicholas, Moli Paul, Diane Purper-Ouakil, Paramala Santosh, Ulrike ME Schulze, Cathy Street, Sabine Tremmery, Frank Verhulst, Dieter Wolke, Swaran P. Singh, MILESTONE Transition Consortium

**Affiliations:** 1 Mental Health and Wellbeing, Warwick Medical School, University of Warwick, Coventry, UK; 2 Coventry and Warwickshire Partnership NHS Trust, Coventry, UK; 3 Health Sciences, Warwick Medical School, University of Warwick, UK; 4 NIHR Policy Research Unit in Mental Health, Division of Psychiatry, University College London, London, UK; 5 Department of Child and Adolescent Psychiatry/Psychology, Erasmus MC-Sophia, Rotterdam, Netherlands; 6 University Hospital Split, School of Medicine University of Split, Croatia; 7 Unit of Epidemiological and Evaluation Psychiatry – UOPEV, IRCCS Centro San Giovanni di Dio Fatebenefratelli, Brescia, Italy; 8 Centre for Health Economics at Warwick, Warwick Medical School, University of Warwick, Coventry, UK; 9 ARQ National Psychotrauma Centre, Diemen, Netherlands; 10 Children’s Health Ireland at Crumlin, SJOG Lucena CAMHS, UCD, Dublin, Ireland; 11 Médecine Psychologique de l’Enfant et de l’Adolescent (MPEA1), Hôpital Saint Eloi, Centre Hospitalier Universitaire de Montpellier, France; 12 Institute of Psychiatry Psychology & Neuroscience, King’s College London, London, UK; 13 Centre for Interventional Paediatric Psychopharmacology and Rare Diseases (CIPPRD), South London and Maudsley NHS Foundation Trust, London, UK; 14 HealthTracker Ltd, Gillingham, Kent, UK; 15 Department of Child and Adolescent Psychiatry, Psychosomatics and Psychotherapy, University Hospital of Ulm, Germany; 16 KU Leuven: Katholieke Universiteit Leuven, Belgium; 17 Erasmus Medical Centre, Netherlands; 18 Department of Psychology, Warwick Medical School, University of Warwick, Coventry, UK

## Abstract

**Background:**

Transition from Child and Adolescent Mental Health Services (CAMHS) to Adult Mental Health Services (AMHS) is poorly managed, with discontinuity of care commonplace, leading to poorer outcomes, while evidence-based interventions to improve transition are scarce. This study is a secondary analysis of the MILESTONE trial, aiming to determine whether managed transition increases the proportion of young people who appropriately transition from CAMHS to AMHS.

**Methods:**

The MILESTONE trial was a multicenter, two-arm, cluster-randomized controlled trial across eight countries at 40 CAMHS sites to compare usual care (UC) to managed transition (MT). MT consisted of training, identification, and assessment of transition readiness and appropriateness. Eligible participants were receiving CAMHS care, IQ ⩾ 70 and within 1 year of their service transition boundary. CAMHS sites were randomized 2:1 between UC and MT. The main outcome was whether participants were receiving care from AMHS at 15 months follow up.

**Results:**

The MILESTONE study included 793 participants, 552 receiving UC and 241 receiving MT. In the MT group, 24.9% transitioned to AMHS at 15 months compared to 14.2% in the UC group (*p* = 0.002), and appropriate transitions (in those with a need for transition at baseline or ongoing clinical need at 15 months) were 32.3% in the MT group compared to 16.4% in the UC group (*p* < 0.001).

**Conclusions:**

A higher proportion of the MT group transitioned to AMHS at 15 months, and there was a higher proportion of appropriate transitions compared to UC. Clinicians and services should consider the incorporation of MT into routine clinical care.

## Introduction

Transition from Child and Adolescent Mental Health Services (CAMHS) to Adult Mental Health Services (AMHS) represents a crucial step at a key time-period for young people experiencing mental health issues. This transition is often poorly planned and executed, resulting in poor experiences (including increased anxiety and uncertainty) for young people and their carers (Appleton et al., 2021; Broad, Sandhu, Sunderji, & Charach, 2017; Singh et al., 2010), and worse outcomes for those who fail to make the transition from child to adult services (Appleton et al., 2019; Islam et al., 2016). There is a high risk of discontinuity of care during this period, and many young people who need continued treatment will ‘fall through the gap’ between CAMHS and AMHS (Singh et al., 2010; Tuomainen, Appleton, & Singh, 2020). Many young patients, for example, are discharged to their GP and subsequently present to mental health services in crisis (Davis & Vander Stoep, 1997; Hovish et al., 2012; Rainer & Abdinasir, 2023).

While the importance of improving young people’s transition has been recognized for many years, research is lacking on how best to manage transition from CAMHS to AMHS and the prospective mental health outcomes of young people who do, and do not, successfully transition (Appleton et al., 2019; Tuomainen et al., 2020).

The aim of the present study is to address these omissions and explore whether the use of managed transition (MT) can increase the proportion of young people making an appropriate transition from CAMHS to AMHS. Specifically, our research questions were:

1. Does managed transition (i.e., training and use of a transition readiness and appropriateness measure) increase the likelihood of transition at 15-month follow-up?
2. Does the effect of managed transition on transition persist following adjustment for potential confounders, including baseline severity, diagnosis, gender, country, and site (Appleton et al., 2023; Gerritsen et al., 2022)?
3. Does managed transition increase the likelihood of appropriate transition (i.e. transition in those with an identifiable need) at 15-months follow up?
4. Does the effect of managed transition on appropriate transition persist when adjusting for the above relevant confounders?

## Methods

### Study design

The present study is a secondary analysis of the MILESTONE trial (Singh et al., 2023); an eight country (Belgium, Croatia, France, Germany, Ireland, Italy, Netherlands, UK), two-arm, cluster-randomized controlled trial (CRCT) to determine the effect of managed transition (MT) compared to usual care (UC) on young people’s outcomes at the transition boundary between CAMHS and AMHS. MT in this instance was a multi-component intervention including the use of the Transition Readiness and Appropriateness Measure (TRAM) (Santosh et al., 2020). The MILESTONE study had strong patient and public involvement embedded throughout, including young advisors, some with experience of transition in mental health services. They provided feedback on the protocol and study documents, designed the intervention leaflets, and advised on recruitment and engagement.

A detailed description of study methodology, including original sample size calculations, is reported elsewhere (Singh et al., 2017). Eligible CAMHS were community teams with a formal upper age limit (transition boundary). The transition boundary was 18 years for the majority of services, but varied according to local practice and formal regulations between 16 and 18 years. Participants were assessed at baseline, which was within 6 months before their transition boundary (T1), 9 months (T2), 15 months (T3), and 24 months (T4) follow-up points. The authors assert that all procedures contributing to this study comply with the ethical standards of the relevant national and institutional committees on human experimentation and with the Helsinki Declaration of 1975, as revised in 2008. All procedures involving human subjects were approved in the UK by the National Research Ethics Service Committee West Midlands – South Birmingham (15/WM/0052) and equivalent ethics boards in the participating countries.

### Participants

The current study included all participants from the MILESTONE cluster-randomized controlled trial (*n* = 793), embedded within the MILESTONE longitudinal study (NCT 03013595) (Singh et al., 2017). Young people were eligible to participate in the trial if they met the following inclusion criteria: (1) a mental disorder defined by DSM-IV-TR, DSM-5 or ICD 10/11; (2) receiving CAMHS care; (3) IQ ⩾ 70; and (4) within 1 year of reaching their service transition boundary (amended in April 2016 to include those up to 3-months older than the transition boundary, as transition decisions were sometimes made after this point). Eligible participants were identified by clinicians and other relevant care staff. Where the young person agreed, their information was passed to a local MILESTONE researcher to provide further information and formally obtain written consent for participation. Participants below the legal age of consent were asked for assent, with their parent/carer providing signed consent (Singh et al., 2017). Written informed consent was obtained from all participants.

### Randomization and masking

Clusters were individual CAMHS sites recruited to the MILESTONE study and 40 sites opened to recruitment for the trial. Individual details of the cluster are given in supplementary material (Supplementary Figure S1 and Table S1). Randomization was 2:1 between UC and MT and was stratified by country, to ensure that the number of clusters per country was divisible by three and that all countries had both control and intervention clusters. All randomizations were conducted by the trial statistician using the statistical software Stata version 14. It was not possible to blind clinicians or researchers to the trial arm. CAMHS sites and study personnel were informed of their allocation after randomization. Participants and parents/carers were only informed of their trial arm if they asked specifically, after consenting to participate.

### Intervention

Managed transition (MT) was a multi-component intervention that included the following:

1. Specific training sessions for clinicians in CAMHS regarding optimal transition and use of the TRAM tool.
2. Systematic identification of participants approaching the transition boundary.
3. A structured assessment of transition readiness and appropriateness (TRAM). Specific versions of the TRAM tool were completed by the young person, a CAMHS clinician, and their parent/carer, if available.
4. Feedback of the TRAM findings to the relevant CAMHS clinician (via the TRAM summary report, see Supplementary data, Figure S2), along with an email advising discussion with the participant and parent/carer and explaining the report and relevant next steps (Supplementary data Figure S3).
5. Following this, the clinician could decide to discuss the recommendations with the young person/carer, call a transition planning meeting, produce a care plan for transition, and prepare the young person for transition if required. In the case of referral to AMHS, the TRAM report should have been attached to the referral letter.

The TRAM tool was developed and validated as part of the MILESTONE study, through existing literature review, expert inputs, and focus groups, and reviewed by MILESTONE’s group of young advisors (Santosh et al., 2020). TRAM was translated into the languages of the eight countries involved in the MILESTONE study, and was available to be completed online via the HealthTracker™ platform (https://www.healthtracker.co.uk), a user-friendly web-based platform allowing measures to be completed remotely (Flamarique et al., 2016). The TRAM summary report presents the scores from the young person, parent/carer and clinician for various items in a user friendly, accessible, visual format, highlighting differences and similarities in scoring and allowing relevant information to be transferred to care plans and referrals (example TRAM summary report, email to clinicians and transition leaflet shown in Supplementary data figures S2–4). Clinicians in the UC group did not receive the TRAM summary report or any training regarding transition, although participants in both arms completed the same assessments.

### Outcome

The primary outcome of interest in the current study is whether the participant was receiving care from AMHS at the 15-month follow-up point of the study (T3) (Singh et al., 2023).

The 15-month follow-up duration was pre-specified as the primary outcome endpoint for the trial (Singh et al., 2017). Prior to this, it was anticipated that many participants would be in the process of transitioning between services, where there can often be delays while patients are discussed between mental health services and responses to referrals are awaited (Appleton et al., 2021). Transition was expected to be complete by 15 months for the majority, given that participants were within one year of their CAMHS transition boundary at entry to the study (Appleton et al., 2019; Singh et al., 2017, 2023). The current study defined transition to AMHS as those participants in current receipt of AMHS care at 15 months. Those receiving other types of care, including those remaining in CAMHS or discharged, were classed as not having transitioned (including those who may have been receiving psychiatric follow-up in private practice).

### Study measures

Participants were assessed at baseline, 9 months and 15 months, either by non-blinded research assistants or self-report measures (Singh et al., 2017). Health of the Nation Outcome Scales for Children and Adolescents (HoNOSCA) was utilized as the primary outcome measure for the trial, which is a widely used, validated, reliable and sensitive global outcome measure indicating severity of mental health problems for child and adolescent mental health and includes domains of behaviour, symptoms, impairments, and social functioning (Garralda, Yates, & Higginson, 2000; Gowers et al., 1999). Total score is a sum of items 1–13, which are each scored on a scale of 0–4 (0 indicating no problem, 4 indicating a severe problem, giving a total range of 0–52). HoNOSCA was rated by a research assistant following semi-structured interviews with the young person, and parent/carer or relevant clinician if available. Current mental health service use was identified through the Sociodemographic and Personal information obtained by participant interviews during the trial at each follow-up point (Singh et al., 2023).

### Statistical analysis

We conducted analysis in the following stages:

1. Unadjusted analyses were conducted using chi-squared tests to determine whether the proportion of participants who transitioned to AMHS at 15 months was greater in the MT than the UC group.
2. Adjusted analyses were conducted by fitting Generalized Linear Mixed Models (GLMM) with a binomial distribution for binary outcomes with a three-level random effects hierarchy. This accounts for clustering of participants within sites and countries, as well as adjustment for relevant fixed effects which are known to affect outcome and transition decisions, such as baseline severity (determined by HoNOSCA total score) (Appleton et al., 2023), diagnosis (Gerritsen et al., 2022), and gender. Due to the heterogenous and polymorphous nature of diagnoses within CAMHS, and the variability in the usage of smaller diagnostic categories across services (O’Connor, Downs, Shetty, & McNicholas, 2020), broad diagnostic categories were chosen for the analysis (S. P. Singh et al., 2023). Additionally, multiple categories of smaller diagnostic groups were likely to lead to convergence issues in statistical models (Anderson & Gerbing, 1984). Consequently, baseline diagnosis was grouped into the five largest categories: mood disorders, anxiety disorders, neurodevelopmental disorders, eating disorders, and other or multiple diagnoses. Models were fitted with transition to AMHS at 15 months (T3) as the dependent variable, with country and site included as random effects and HoNOSCA total score at baseline, gender, diagnostic group and treatment group (MT vs US) as fixed effects (utilizing the same analytic approach for primary outcome in the MILESTONE cluster randomized controlled trial, (Singh et al., 2017, 2023).
3. For further analyses, participants were separated into those with and without a ‘transition requirement’. Transition requirement was defined as those who either:
  - Received a recommendation to transition from CAMHS to AMHS during the baseline TRAM assessment by a CAMHS clinician, or;
  - Had an ongoing clinical need at 15 months (T3). Ongoing clinical need in the study was defined as a score of 2 or above on individual HoNOSCA items within psychiatric symptom domains (Question 1–4 behavioral subscale, questions 7–9 symptoms subscale, and question 11) (Appleton et al., 2023; Burgess et al., 2009).
4. Unadjusted analyses using Chi-squared tests were repeated among subgroups where participants were separated into those with and without a ‘transition requirement’. This aimed to determine whether the proportion of participants who had an ‘appropriate transition’ to AMHS at 15 months (i.e. those who had a transition requirement and were receiving care in AMHS at 15 months) was greater in the MT than the UC group.
5. Finally, the same models described above in point 2 were repeated for subgroups of participants with and without a ‘transition requirement’, to determine whether managed transition had a differential effect on appropriate transition to AMHS while adjusting for relevant fixed and random effects. Further subgroup analysis was repeated after removing participants who remained in CAMHS at 15 months (i.e. only including those who either transitioned to AMHS or were discharged from mental health services). Unadjusted analyses using Chi-squared tests and GLMMs as described above were repeated for this subgroup using the same fixed and random effects.

All analyses were on an intention-to-treat basis and were conducted in IBM SPSS Statistics for Windows, version 29.0 (IBM Corp, 2023).

## Results

### Participants

**Figure 1.**
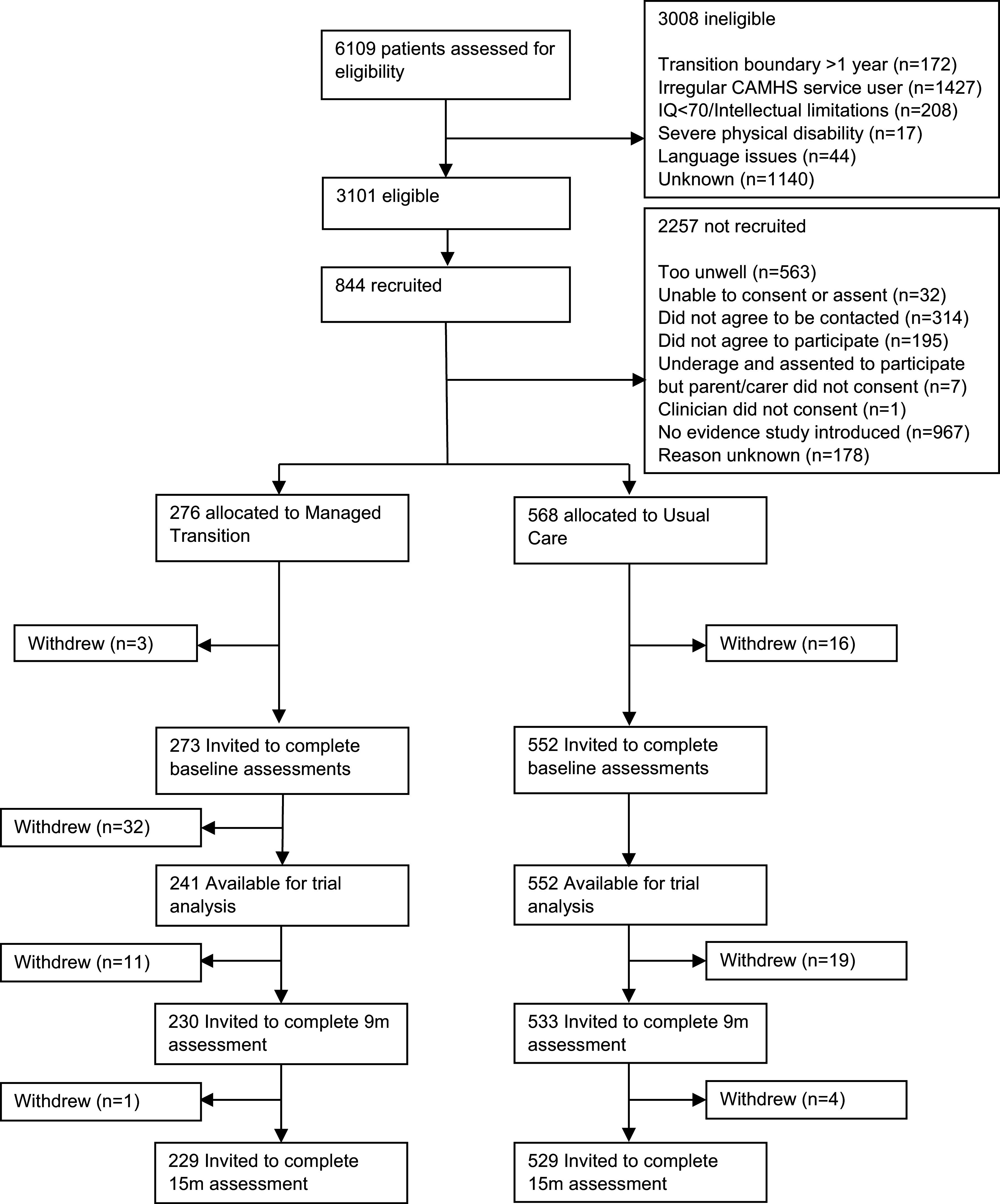
Trial profile and patient flow.

Baseline characteristics of participants are shown in Table 1. In total, 844 participants were recruited between October 1, 2015 and December 31, 2016. Details of participants flow through the trial, CONSORT diagram, reasons for withdrawal, adverse events, and details of participating CAMHS clusters are reported previously (Singh et al., 2023) and detailed in Figure 1 (see also Supplementary Data Figure S1, Table S1). Briefly, 19 participants withdrew before baseline assessment and 32 were withdrawn from a single site in Croatia due to uncertainty concerning the validity of participant consent. Therefore, 793 participants were available for analysis. As shown in Table 1, baseline TRAM recommendations and the presence of ongoing clinical need at T3 were very similar between groups, with 21% participants in total receiving a recommendation to transition to AMHS, and 71.4% demonstrating an ongoing clinical need at T3. Overall, 79.4% of participants demonstrated a transition requirement (i.e. ongoing clinical need at T3 or TRAM recommendation for transition to AMHS), whereas 20.6% had no transition requirement (i.e. no ongoing clinical need and no TRAM recommendation for transition to AMHS). The proportion of participants with a transition requirement was also similar between trial arms (Table 1).

**Table 1.** Baseline characteristics of total sample

	UC (*n* = 552)	MT (*n* = 241)	Total (*n* = 793)
Age (mean, s.d.)	17.48 (0.59)	17.64 (0.51)	17.53 (0.57)
Gender (*N*, %)			
Female	340 (61.59)	149 (61.83)	489 (61.66)
Male	211 (38.22)	90 (37.34)	301 (37.69)
Prefer not to say	1 (0.18)	2 (0.83)	3 (0.38)
TRAM recommendation for AMHS^a^ (*N*, %)			
AMHS recommended	99 (21.2)	45 (20.5)	144 (21)
Other recommendation	368 (78.8)	174 (79.5)	542 (79)
HoNOSCA clinical need T3^b^ (*N*, %)			
Clinical need	284 (71.5)	122 (70.9)	406 (71.4)
No clinical need	113 (28.5)	50 (29.1)	163 (28.6)
Transition required (TRAM recommendation or clinical need) total^c^	327 (79.8)	139 (78.5)	466 (79.4)
Transition not required (TRAM did not recommend AMHS and no clinical need)^c^	83 (20.2)	38 (21.5)	121 (20.6)
MH care received at T3^d^ (*N*, %)			
AMHS	56 (14.2)	44 (24.9)	100 (17.5)
CAMHS	88 (22.3)	38 (21.5)	126 (22.1)
Discharged	250 (63.5)	95 (53.7)	345 (60.4)
Diagnosis (*N*, %)			
Anxiety disorders	69 (12.5)	25 (10.4)	94 (11.9)
Mood disorders	104 (18.8)	60 (24.9)	164 (20.7)
Neurodevelopmental disorders	186 (33.7)	71 (29.5)	257 (32.4)
Eating disorders	45 (8.2)	9 (3.7)	54 (6.8)
Other^e^	148 (26.8)	76 (31.5)	224 (28.2)

a Missing = 107,

b Missing = 224,

c Missing = 206,

d Missing = 222,

e Other diagnoses include – substance related disorders, psychotic disorders, obsessive-convulsive and related disorders, trauma and stress related disorders, dissociative disorders, somatic symptoms and related disorders, disorders of adult personality and behaviour, gender dysphoria, unspecified disorder and multiple primary diagnoses.

### Transition to AMHS at 15 months

Table 2 shows the proportion of participants in MT and UC groups who had transitioned to AMHS at T3 (15 months). In the MT group, 24.9% transitioned to AMHS at T3 compared to 14.2% in the UC group (*p* = 0.002). In the subgroup analysis within the group with a ‘transition requirement’, 32.3% within the MT group transitioned to AMHS at T3 compared to 16.4% in the UC group (*p* < 0.001), whereas there was no significant difference in transition between MT and UC in the group without a ‘transition requirement’. For further subgroup analysis after removing those who remained in CAMHS at T3, the proportion who transitioned to AMHS was 31.7% (MT) compared to 18.3% (UC), *p* = 0.002, and among those with a ‘transition requirement’ the proportion who transitioned to AMHS was 40.6% (MT) and 21.4% (UC), *p* < 0.001.

**Table 2.** Transition to AMHS at 15 months

	UC	MT	P value
Transition to AMHS at T3, *n* (%)^a^	56 (14.2)	44 (24.9)	**0.002**
AMHS T3 where transition required^b^	46 (16.4)	41 (32.3)	**<0.001**
AMHS T3 where no transition required^b^	7 (8.8)	0 (0)	0.075

a Total = 793, missing = 222,

b Total = 587, missing = 66. Chi squared test.

### Multilevel adjusted analysis

Table 3 shows the adjusted analyses using binary logistic mixed-effect models. Within the whole group, the treatment intervention (MT) led to a slightly increased probability of transition to AMHS at T3 after adjusting for fixed and random effects (estimated OR 1.56, 95% CI 0.84–2.91), though this was not statistically significant (*p* = 0.158). The adjusted intraclass-correlation coefficient for transition to AMHS at 15 months in the same country was 0.033 (95% CI 0.005–0.184), and in the same cluster and country was 0.033 (95% CI 0.007–0.137). In the subgroup analysis, within the group with a ‘transition requirement’, MT increased the odds of transition to AMHS at T3 (estimated OR 2.09, 95% CI 1.05–4.17), which was statistically significant after adjustment for fixed and random effects (*p* = 0.036). Within the group without a ‘transition requirement’, there was no significant effect of managed transition on the odds of transition at T3. For further subgroup analysis after those who remained in CAMHS at T3 were removed, the results were similar, with no overall effect on transition to AMHS at T3 (estimated OR 1.43, 95% CI 0.76–2.68, *p* = 0.264). However, the effect of MT in those with a ‘transition requirement’ within this subgroup remained significant with a similar effect size (estimated OR 2.1, 95% CI 1.04–4.24, *p* = 0.038).

**Table 3.** Multilevel binary logistic model with three levels (country, site, participant)

	Effect estimate OR for MT	95% CI	*p* value
Transition to AMHS at T3	1.563	0.84–2.906	0.158
AMHS T3, where transition is required	2.089	1.048–4.165	**0.036**
AMHS T3, where no transition is required	0.287	0.031–2.628	0.266

Treatment group (MT vs UC) as dependent variable.

Adjusted for gender, baseline HoNOSCA and diagnostic group as fixed effects, along with treatment group (MT vs UC) as independent variable. Country and site were included as random effects.

## Discussion

### Main findings and implications

The results of the present study demonstrate that the use of MT leads to a higher proportion of participants transitioning to AMHS compared to UC. While this difference was not statistically significant across the whole trial after adjusting for potential confounders, in secondary analyses, the subset of participants with a ‘transition requirement’ was twice as likely to transition to AMHS in the MT group compared to UC. There was no such increase in the group without a transition requirement. This suggests that the intervention of managed transition led to a greater proportion of appropriate transitions to AMHS (i.e. in those with a pre-identified need for transition at baseline, or ongoing clinical need) without leading to an overall increase in inappropriate transitions in those without the need for AMHS. In fact, in the MT intervention arm, there were no transitions to AMHS among the group without a transition requirement (i.e. no inappropriate transitions). The lack of increased transitions in the group without a ‘transition requirement’ suggests that the MT intervention did not increase transitions universally. This suggests that through completion and discussion of the TRAM summary report, subsequent transition planning meetings with participants and carers, a decision was reached about the need for transition to AMHS, which was able to correctly identify ‘appropriate transitions’ more readily compared to usual care. This also appeared to reduce the need for onward referral where transition to AMHS may have been unnecessary, possibly due to clear communication and a patient-centered approach to transition decisions, which are often lacking in routine care (Appleton et al., 2021; Broad et al., 2017).

After removing participants who remained in CAMHS beyond the transition boundary, there was no difference to the overall findings, i.e. those in the MT group with a transition requirement were significantly more likely to transition to AMHS compared to UC.

This cluster RCT was the first trial to have assessed the effectiveness of an intervention on improving transition between CAMHS and AMHS (Appleton et al., 2019; Paul, Street, Wheeler, & Singh, 2015), and has previously demonstrated that the use of MT led to improved mental health of young people after 15 months (Singh et al., 2023). The MT model implemented within the trial utilized structured assessments of transition need, readiness, and appropriateness, and enabled shared decision making between the young person, parent or carer, and CAMHS clinician, allowing young people to be closely involved in both the decision and preparation for transition from CAMHS to AMHS (Singh et al., 2017). This addresses many of the issues identified by young people, parents, and carers with the current process of transition from CAMHS to AMHS, including lack of preparation, discontinuity, and lack of patient and carer involvement (Boonstra et al., 2024; Broad et al., 2017; Street et al., 2023). The nature of the intervention is such that it is easy to implement and incorporate into routine care, without significant reorganization of services and their associated challenges (Maxwell et al., 2019; McGorry, Bates, & Birchwood, 2013).

Previous analysis of associated costs from the MILESTONE Study showed the MT intervention was relatively inexpensive, with direct intervention delivery costing €17–65 per young person, and training costs ranging from €22–176 (Singh et al., 2023). However, subsequent economic analysis of the MILESTONE data has demonstrated that although service costs fell by 50% from pre to post service transition boundary, those who remained engaged with mental health services accrued the greatest costs, particularly so for those who were more unwell at baseline (Canaway et al., 2023). This suggests that a greater rate of transition to AMHS reported here could potentially lead to higher healthcare costs in the short term. Notwithstanding, the findings of the present study that the proportion of those who transitioned to AMHS at 15 months increased principally in those with an identified need may suggest that MT leads to more efficient allocation of ongoing resources (e.g. through reducing long-term need for specialist service and inpatient care, which are particularly costly), potentially offsetting these healthcare costs over time in addition to benefits of wider societal costs (Barr et al., 2017). The provision of appropriate treatment early on may allow savings in terms of physical and mental health and wider societal costs in the future (Children’s Commissioner, 2025), though this has not been formally evaluated for this intervention. Longer term impacts on costs and service utilization would be an important area of further research when considering the implementation of interventions such as MT into routine clinical practice.

### Strengths and limitations

This study is the first RCT to investigate the effect of a specific intervention on the transition from CAMHS to AMHS. The study was conducted across eight European countries and demonstrated good recruitment and retention and is highly generalizable across a range of mental health services.

Nonetheless, there are some limitations that should be considered in the interpretation of the results. There were missing data for participants at 15 months for those who had dropped out of the study. Due to the population of young people who are highly mobile, likely to move for higher education, training or employment, some loss to follow up was broadly as anticipated (Singh et al., 2017). However, it is possible that the most severely ill participants were under-represented in the study, and they may have been more likely to drop out. This group is also more likely to require ongoing care in AMHS. Comparing participants for whom there were any missing data at 15 months with those with complete data, there were no significant differences in age, gender, baseline HoNOSCA total score, diagnosis, or trial arm between those who dropped out and those who remained in the study (Supplementary data Table S2).

The trial was unblinded to participants, clinicians, and assessors due to the nature of the intervention. Furthermore, although clinicians in the UC arm did not receive the TRAM summary report or any associated training, they did complete the same assessments, including the TRAM clinician report, which, along with knowledge of participation in the trial, may have influenced decision making. The TRAM clinician report and recommendation were completed by CAMHS clinicians for 86.5% of participants, indicating a relatively good level of adherence. However, the trial did not record to what extent the MT intervention was adhered to within participating sites, and it is therefore not possible to state whether discussion of the report with the participant, arranging transition meetings, and attaching the TRAM summary report to subsequent referrals was adhered to as was suggested in the MT training.

The current analysis assessed only the transition to AMHS at 15 months, which was established as the optimal time point to determine the trial’s primary outcome. Before this, many participants were still in the process of transitioning, whereas by 15 months, the transition was expected to be complete for most (Singh et al., 2017, 2023). However, a significant proportion of participants (22.1%) remained in CAMHS at 15 months, beyond the service transition boundary, though the effect of MT remained the same when comparing only those who either transitioned to AMHS or were discharged. Though similar proportions have been observed in other studies (Appleton et al., 2019), this represents a variation in practices across countries (Signorini et al., 2017) and may also represent the challenge for clinicians to discharge patients where there are concerns that either AMHS or primary care may not be able to meet their needs (Reneses et al., 2023; Shahid, De Simone, Appleton, & Bisp, 2025). Moreover, due to the relatively short period of follow-up after transition to AMHS, it was not possible to determine whether there were further improvements in clinical outcomes for those who transitioned to AMHS, and whether the MT intervention led to greater ongoing improvements compared to usual care. Additionally, a small group of participants were discharged and subsequently returned to care either in CAMHS or AMHS (Gerritsen et al., 2022) over the course of the study. Further research is needed to determine the effect of interventions such as MT on this group of patients and whether improvements in clinical outcomes are maintained post-transition.

## Conclusions

The use of MT led to a higher proportion of patients within the study transitioning to AMHS by 15 months. However, this difference was not statistically significant in the adjusted analyses using mixed-effect models. In secondary analyses, among participants with an identified ‘transition requirement’, and after adjusting for other relevant baseline variables, there was a significant difference between treatment groups: those in the MT group were twice as likely to be receiving care from AMHS at 15 months compared to those in the UC group. Moreover, there were no transitions to AMHS among the group without a transition requirement. This indicates that MT can help ensure the young people who require ongoing care transition to AMHS, which may lead to optimal allocation of healthcare resources. Clinicians and services should consider the incorporation of the TRAM tool into routine clinical care.

## Supporting information

10.1017/S0033291726103766.sm001 Rowland et al. supplementary material Rowland et al. supplementary material

## Data Availability

Requests for original (fully anonymized) participant data may be made to the corresponding author.

## Supplementary material

The supplementary material for this article can be found at http://doi.org/10.1017/S0033291726103766.
