# Supplementary material for: The effect of managed transition on the proportion of young people transitioning from CAMHS to AMHS: Analysis of the Milestone Cluster Randomised Clinical Trial
Source: Psychol Med. 2026 Apr 6;56:e91. doi: 10.1017/S0033291726103766 (PMC13079206; doi:10.1017/S0033291726103766)
Supplement: Rowland et al. supplementary material [file S0033291726103766sup001.docx]

## Figure S1: Recruitment and allocation of clusters

Footnote: For the RCT, 45 clusters were randomised 2:1 between control and intervention. For the combined RCT and longitudinal cohort study (58 clusters), this equates to a randomisation ratio of roughly 3:1 (43 control and 15 intervention). The longitudinal study (not reported here) comprises the 13 clusters directly allocated plus the 30 usual care (control) clusters from the RCT.

## Figure S2: Example of TRAM summary report

## **Figure S3: Email to clinicians that accompanied the TRAM** summary **report**

**Dear Dr Smith,**

We are very grateful for your participation in the MILESTONE study.

Please find attached the **TRAM (Transition Readiness and Appropriateness Measure) score summary report** related to your patient/client [First name, Last name **or** patient’s hospital/clinic ID]. It contains

a) items linked with [his/her] appropriateness for transition to adult mental health services, which are relevant to your transition decision making; and

b) items indicating [his/her] readiness for transition, which may need to be addressed by clinicians on either side of the transition boundary – in order to facilitate a smoother transition.

**Page 1** contains clinician guidance to the score summary report. It explains the data presented in the graphs (PLOTS) and tables on pages 1-2.

**Page 2** presents three plots summarising symptoms, risk factors and overall disruption experienced by the young person as rated by the young person, parent/carer and clinician; and a fourth plot summarising potential barriers to a smooth transition for that YP.

**Page 3** contains all young person, parent/carer and clinician scores from the TRAM in tabular format, with high scores (rated moderate or higher) highlighted in yellow.

In order for you to make the most of the information contained in this report, we recommend the following:

1) Note high scores, or those that stand out, in PLOTS 1-3 as they indicate items that may benefit from consideration when deciding on transition pathways (This information may also be inferred from tables 1, 3 and 4 in Part A).
2) In PLOT 4, note the type and level of potential barriers to successful transition. These are key areas that need to be worked on to ensure successful transition (table 1, Part B).
3) Note the tables that do not have a corresponding graph:
   a. Overall illness (table A2)
   b. Factors that may be affecting symptoms, e.g. drug and alcohol misuse (table A5).
   c. Other life changes (table 2, Part B), which may need consideration when working on potential barriers to a smooth transition.
4) **Note the differences in responses, if any, by the young person, parent/carer and clinician in the graphs and tables.** Differences of opinion are often not spoken out loud and there is potential to address them in a solution-focused way.
5) Use the findings from the TRAM score sheet to focus conversation with your patient/client and their parent/carer on issues surrounding end of care at CAMHS and potential transition to AMHS or other community based service. The TRAM score sheet is confidential so please don’t provide them with copies of it. Instead, use the scores to check understanding of issues and what your patient/client thinks should be done next.
6) Add critical items from the score sheet (items with high score) to a transition plan.
7) Design goals for critical items that are achievable.
8) Include the TRAM summary score sheet in your patient’s/client’s medical notes.
9) If transition to AMHS is decided upon, please include the TRAM summary score report in the referral letter and include our letter to the AMHS clinician (attached to this communication [Appendix 2]). The letter offers the opportunity to discuss the report with one of the MILESTONE team members.

The enclosed “MILESTONE transition leaflet” provides further guidance on factors associated with good quality transition, and contains a frequently asked questions section about TRAM which you may find helpful.

We would like the TRAM score summary report to be as clear to you as possible, so you can make the most of it when making the decision about transition and acting upon it. I am happy to come and explain it further, so please let me know of a date and time most suitable for you. We can also discuss over the phone, if the information in the TRAM summary is unclear or difficult to interpret. Please call me on [tel.no], or email me on [email address] to arrange a suitable time.

I look forward to hearing from you. With best wishes, [MILESTONE research assistant]

## **Figure S4: Transition leaflet that accompanied the TRAM** summary **report**

## Table S1: Characteristics of participating CAMHS (clusters)

| **Country** | **Site** | **Service boundary**  **(age in years)** | **Written transition policy in place? (Y/N)** | **Type of service** |
| --- | --- | --- | --- | --- |
| Belgium | Antwerp  Brugge  Brussel  Genk  Leuven  Kortenberg | 18  18  18  18  18  18 | Unknown | Hospital based  Hospital based  Community based  Community and Hospital based  Hospital based  Community and Hospital based |
| Croatia | Osijek  Split  Zagreb | 18  18  N/A | N/A | Other  Other  Withdrew |
| France | Marseille  Montpellier  Tours | 18  18  17 | Y | Community and Hospital based  Community and Hospital based, Other  Hospital based |
| Germany | Augsburg  Ravensburg  Ulm | 18  18  18 |  | Hospital based  Hospital based  Hospital based |
| Italy | Bari  Brescia  Lecco  Milano SP  Modena  Monza  Padova  Parma  Roma^2^ | 18  18  18  18  18  18  18  18  18 | Y  Y  Y  Y  Y | Hospital based  Community and Hospital based  Community and Hospital based  Community and Hospital based  Community and Hospital based, Other  Community and Hospital based  Community and Hospital based  Community based, Other  Hospital based |
| Netherlands | Erasmus MC  GGZ WNB^1^  Lucertis  Mondriaan  Virenze  Yulius B | 18  18  18  18  18  18 | Y | Hospital based  Community based  Community and Hospital based  Community based, Other  Community based  Community based |
| Republic of Ireland | North Cork  Roscommon/East Galway  South Lee/ West Cork | 18  N/A  18 |  | Community based  Community based (Withdrew)  Community based |
| United Kingdom | Dudley  Lambeth  Lewisham  North Worcestershire  South Staffordshire and Shropshire  Southwark  Wolverhampton | 16  18  18  18  18  18  18 | Y  Y  Y  Y | Community based  Community and Hospital based  Community and Hospital based  Community based  Community based  Community and Hospital based  Community based |

^1^ Geestelijke Gezondheidszorg Westelijk Noord-Brabant

^2^ Privately funded, all other government and/or health insurance funded

**Table S2: Comparison of baseline characteristics between those with missing data at T3 (15 months)**

|  | Full data present at T3 (*n* = 521) | Missing data at T3^a^  (*n* = 272) | P-value |
| --- | --- | --- | --- |
| Age (mean, s.d.) | 17.5 (0.61) | 17.6 (0.49) | 0.070 |
| Gender (*N*, %) | | | |
| Female | 337 (64.7) | 152 (55.9) | 0.052 |
| Male | 182 (34.9) | 119 (43.8) |  |
| Prefer not to say | 2 (0.4) | 1 (0.4) |  |
| HoNOSCA baseline (mean, S.D) | 11.8 (6.65) | 12.8 (7.56) | 0.063 |
| RCT intervention (*N, %)* | | | |
| Managed Transition | 161 (30.9) | 80 (29.4) | 0.670 |
| Usual Care | 360 (69.0) | 192 (70.6) |  |
| Diagnosis (*N,* %) | | | |
| Anxiety disorders | 69 (13.2) | 25 (9.2) | 0.394 |
| Mood disorders | 102 (19.6) | 62 (22.8) |  |
| Neurodevelopmental disorders | 172 (33.0) | 85 (31.3) |  |
| Eating disorders | 36 (6.9) | 18 (6.6) |  |
| Other | 142 (27.3) | 82 (30.1) |  |

^a^Participants for whom T3 data was missing for any variable used in analysis in the current study

Independent samples T-test used to compare continuous variables (age, HoNOSCA)

Chi-squared tests used to compare categorical variables (Gender, Treatment Group, Diagnosis)
